# Enhancing SARS-CoV-2 surveillance through regular genomic sequencing is an essential element of COVID-19 control in resource-limited settings

**DOI:** 10.11604/pamj.2022.41.88.31821

**Published:** 2022-02-01

**Authors:** Grant Murewanhema, Faith Mutsigiri-Murewanhema, Edward Kunonga

**Affiliations:** 1 Faculty of Medicine and Health Sciences, University of Zimbabwe, Harare, Zimbabwe,; 2 School of Health and Life Sciences, Teesside University, Middlesbrough, England

**Keywords:** SARS-CoV-2, herd immunity, variants of concern, genomic sequencing

## To the editors of the Pan African Medical Journal

Vaccination is a key intervention for SARS-CoV-2 control [1]. Emerging evidence has shown the benefits of the different SARS-CoV-2 vaccines in reducing the risk of death, severe disease, hospitalization, mild and moderate disease, and incident infections [2]. Attaining herd immunity (HI) is a critical public health goal. The herd immunity threshold (HIT) is the proportion of the population that needs to become immune to an infectious agent, in this case, SARS-CoV-2, to the extent that non-immune people are unlikely to interact with an infected person [3]. At this point, population-level reduction of breakthrough infections is realized, leading to the protection of vulnerable populations not eligible for vaccination. The HIT is a function of the effective reproduction number (R0) and is estimated to be between 70-90% for SARS-CoV-2; however, it is also variant-dependent [3].

Attaining HI through vaccination depends on the success of vaccination programs. Unfortunately, several threats to success exist, more pronounced in the low-and-middle-income countries (LMICs), especially Sub-Saharan Africa (SSA) [4]. These include vaccine hesitancy, vaccine nationalism and inequitable distribution and access to vaccines, especially the mRNA-based vaccines [4-6]. Some African countries outside of the COVID-19 Vaccines Global Access (COVAX) initiative, such as Zimbabwe, had to seek alternative sources of vaccines, such as Russia, China and India [7]. The emergence of variants of concern (VOCs) poses a big threat to the attainment of HI through shifts in vaccine effectiveness and HIT [8]. Therefore, surveillance for VOCs through regular genomic sequencing is important to inform vaccination programs globally [8]. In this scope, we highlight the importance of this critical aspect of surveillance, and the need to build local capacity for genomic sequencing in LMICs.

Since the onset of the pandemic, several VOCs have emerged, including the alpha, beta, gamma, and more recently, the delta variants [2]. Evidence showed reduced effectiveness of the Pfizer-BioNTech, Janssen and AstraZeneca vaccines against the beta variant compared to the ancestral wild type; similarly, the effectiveness against the delta variant could be reduced [2]. With the emergence of new VOCs, it will be difficult for countries to attain HITs for the emerging variants, as this shift, and depending on the extent of mutations, some emerging variants may come with major structural changes to the immunogenic epitopes that render the existing vaccines ineffective [8]. Random genomic sequencing in Zimbabwe showed that the beta variant was responsible for over 70% of the cases in December 2020, and over 95% in January 2021 [9]. Similarly, the delta variant was reported as responsible for 98% of the cases in June-July 2021. Without regular genomic sequencing, it is likely that these variants may go undetected, especially in LMICs, where this is easily available [8].

Zimbabwe had to take its specimens to the Quadram Institute in the UK for genomic sequencing, which is not perceived as a priority in countries that struggle with regular real-time polymerase chain reaction (RT-PCR) testing for SARS-CoV-2. LMICs must prioritize adequately investing in capacity building for SARS-CoV-2 testing as part of enhancing surveillance [10]. Surveillance is critical for epidemic control and facilitates detection of changing epidemiological trends, timely diagnosis, treatment and isolation of confirmed cases as well as contact tracing and quarantining of contacts to break chains of transmission [10]. Additionally, LMICs must invest in genomic sequencing as part of regular surveillance, to facilitate timely detection of emerging VOCs. This is also important for informing the effectiveness of ongoing vaccination programs. Collaborating with academic research institutions in developed countries provides an opportunity for genomic sequencing capacity building.

Ensuring optimal observation of infection prevention and control (IPC) strategies in countries that are lagging behind in terms of vaccination programs remains important. The rising incidence of breakthrough infections in countries at advanced stages of vaccination indicates the need to maintain these IPC measures globally for now [2]. Emerging VOCs and escape mutations provide plausible explanations for breakthrough infections in the vaccinated cohorts [8]. Fortunately, case fatality has been low among the vaccinated, pointing towards protection against severe disease and mortality despite the breakthrough infections [2]. Despite emerging evidence of reduced vaccine effectiveness against the delta variant, countries must vaccinate adequately with the currently available vaccines, as they still confer reasonable protection against adverse disease outcomes [2].

## Conclusion

Relevant stakeholders in LMICs must devise ways of enhancing surveillance for SARS-CoV-2 including building capacity for local genomic sequencing through collaborative partnerships. This is important for monitoring epidemiological trends, timely detection of VOCs and informing vaccine development. Local development of effective vaccines would be the greatest next step. Unfortunately, the majority of LMICs lack the capacity to manufacture vaccines and must rely on imports for now.
